# Reconstruction of necrotizing soft tissue infection in the auricle and temporal region: a case report

**DOI:** 10.1080/23320885.2022.2157281

**Published:** 2022-12-22

**Authors:** Junpei Saito, Shoichi Ishikawa, Shigeru Ichioka

**Affiliations:** Department of Plastic and Reconstructive Surgery, Saitama Medical University, Saitama, Japan

**Keywords:** Necrotizing soft tissue infection (NSTI), auricle and temporal region, temporalis muscle flap, creation of an auriculotemporal sulcus

## Abstract

A 43-year-old female patient had a necrotizing soft tissue infection in the temporal region. Because of the necrotic temporoparietal fascia, auricular reconstruction was attempted using the temporalis muscle flap; the flap was successfully placed. The use of the temporalis muscle flap was considered a treatment option for salvaging the auricle.

## Introduction

Necrotizing soft tissue infection (NSTI) is a rapidly progressive cutaneous soft tissue infection with a high mortality rate. It most commonly occurs in the perineal area and lower extremities [1,2] but rarely in the head and face region with a high mortality rate [3]. Immediate debridement of the infected and necrotic tissues is necessary to save lives; however, the tissue loss is often extensive and reconstruction is difficult. In this report, we describe a case of NSTI in the auricle and temporal region, a rare site of onset, in which the auricle was salvaged and treated with the temporalis muscle flap.

## Case presentation

A 43-year-old woman presented to our department with chief complaints of pain and skin ulceration around the right auricle. At 13 days before presentation, the patient was diagnosed with herpes zoster at the first branch of the right trigeminal nerve.

**Figure 1.**
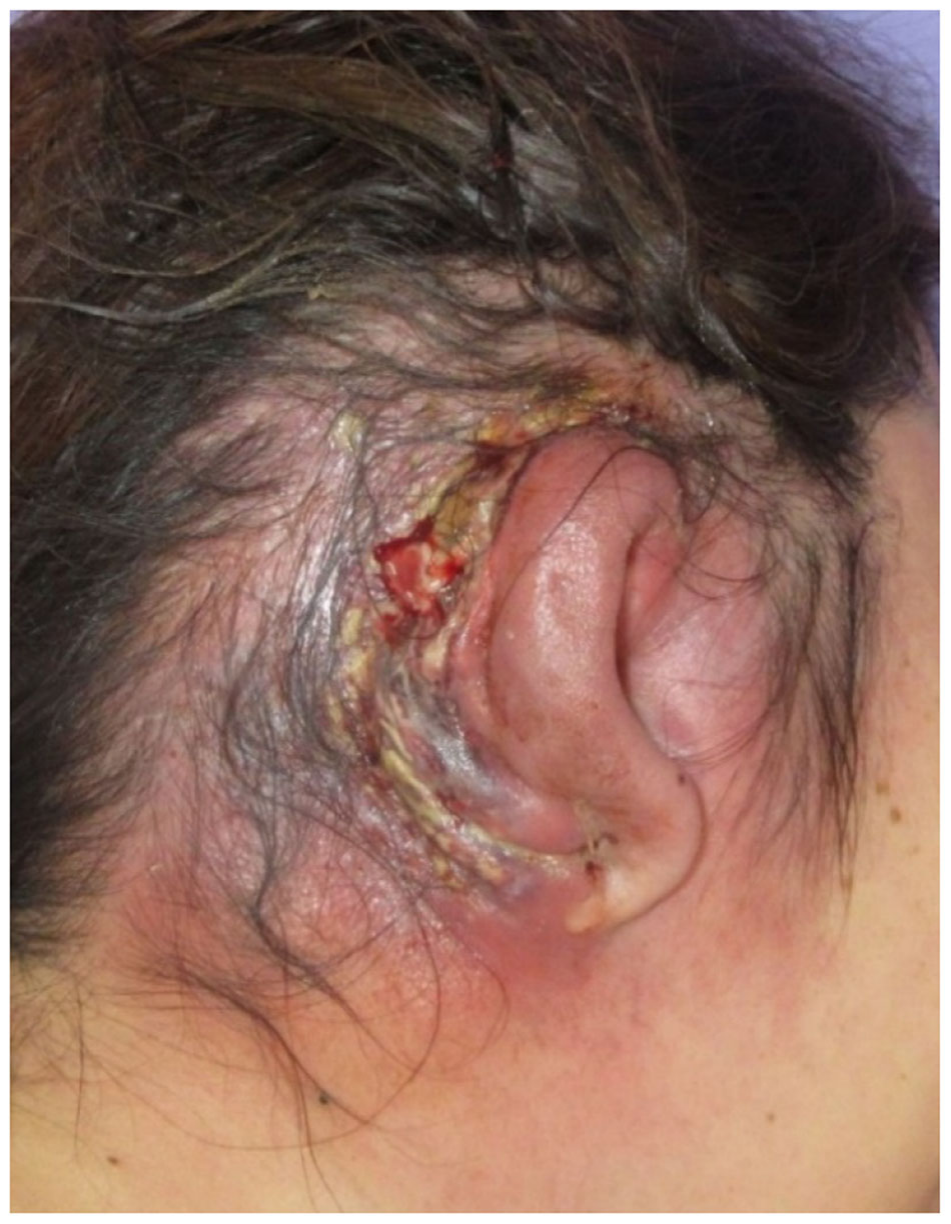
The first visit. Redness, swelling, necrosis, and pus in the right ear.

During the first hospital visit, skin and subcutaneous tissue necrosis and pus drainage were mainly observed on the posterior surface of the right auricle. The right auricle and the surrounding temporal area were erythematous and swollen (Figure 1). Vital signs were as follows: clear consciousness, heart rate of 118/min, blood pressure of 151/103 mmHg, a body temperature of 37.1 °C, and respiratory rate of 20/min. Blood tests showed leukocytes of 26,490/μl; CRP, 40.97 mg/dl; Hb, 14.7 g/dl; Na, 127 mEq/l; creatinine, 0.43 mg/dl; blood glucose, 475 mg/dl; HbA1c, 12.1%; high inflammatory response; and poor glucose control. The laboratory risk indicator for necrotizing fasciitis score was 9 points. The patient reported that she had no history of diabetes mellitus, but a slowly progressing insulin-dependent diabetes mellitus was diagnosed; during hospitalization, the diabetologist administered insulin to control her blood glucose level. Blood and pus cultures revealed methicillin-susceptible *Streptococcus aureus.* Antibiotic treatment was started with meropenem and daptomycin. Meropenem was administered for 6 days and daptomycin for 4 days. This regimen was subsequently de-escalated to cefazolin. Antibiotics were administered for 22 days, at which point acute signs of infection had subsided.

**Figure 2.**
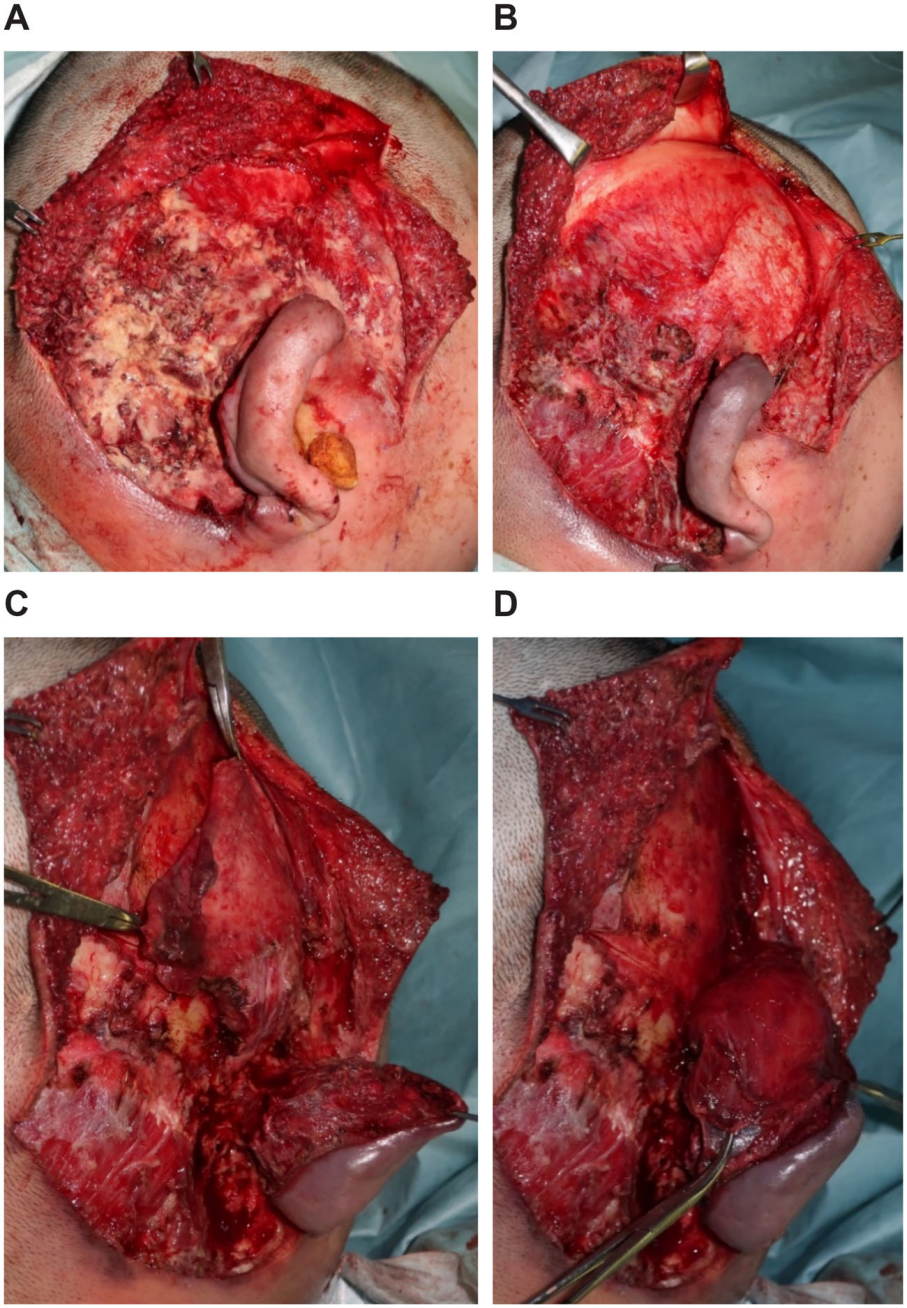
First surgery. (A) Necrosis from the subcutaneous tissues to the temporal fascia. (B) After the necrotic tissue excision, the temporalis muscle is exposed. (C) Creating the temporalis muscle flap. (D) The temporalis muscle flap is covered over the detached auricle.

**Figure 3.**
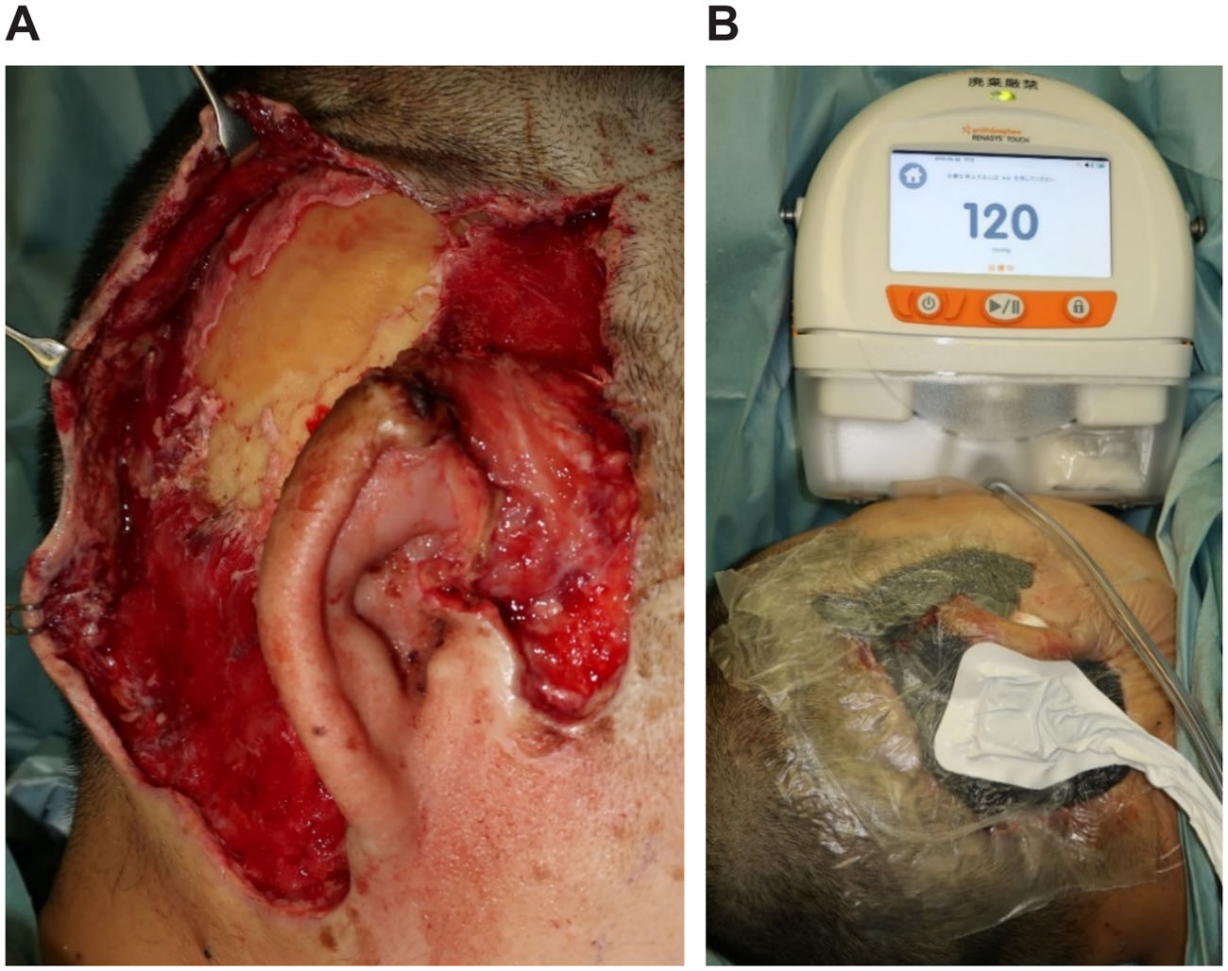
Second surgery, 12 days after. (A) After debridement, the extracranial plate is exposed. (B) Applying NPWT (RENASYS⋄ TOUCH, Smith & Nephew) with a negative pressure of −120 mmHg.

**Figure 4.**
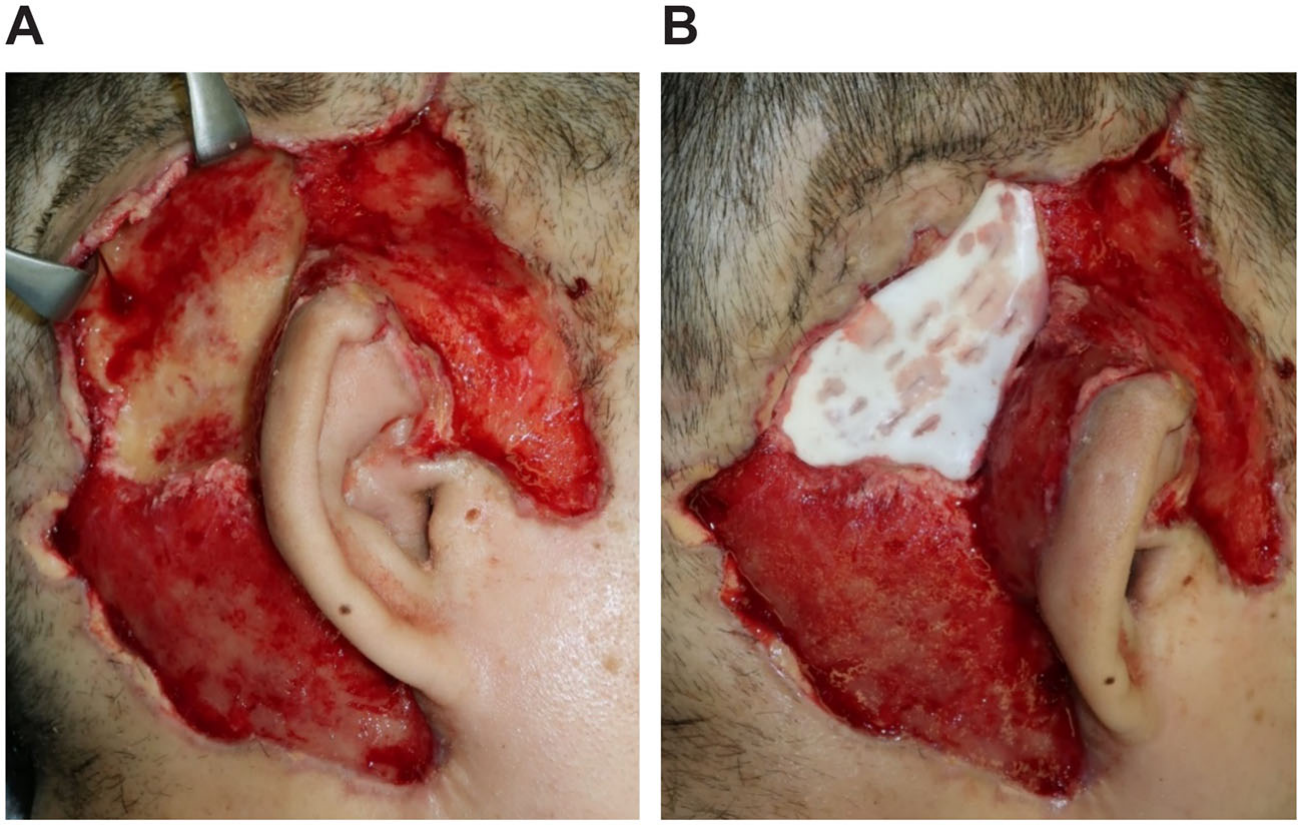
Third surgery, 26 days after. (A) The extracranial plate is removed to the depth where petechial hemorrhage. (B) Artificial dermis (TERUDERMIS^Ⓡ^, Olympus Terumo Biomaterials Corp) is placed.

An emergency debridement was performed. The necrotic skin was excised, and additional skin incisions were made so that the necrosis of the subcutaneous tissues could be confirmed. The necrotic subcutaneous tissue and temporal fascia were excised one layer at a time. Visually, the temporalis muscle did not appear to have obvious necrosis. Furthermore, the upper portion of the auricle was detached by debridement, and a color change occurred due to insufficient blood flow. To salvage the auricle, the temporalis muscle flap was elevated above the periosteum and inverted to cover the auricle (Figure 2). A second debridement was performed 12 days later, and the necrotic skin and periosteum were excised. Negative-pressure wound therapy (NPWT) was started at the end of surgery (Figure 3). A third debridement was performed 26 days after. Because of the exposed temporal bone, the extracranial plate was shaved until petechial hemorrhage could occur, and the artificial dermis was applied (Figure 4). NPWT was used for 9 days to fix the artificial dermis. When purulent exudation and edematous granulation increased, local treatment consisted of washing and dressing to achieve wound bed preparation and good granulation of the ulcer surface. The artificial dermis was successfully applied, and the skull was no longer exposed due to granulation.

**Figure 5.**
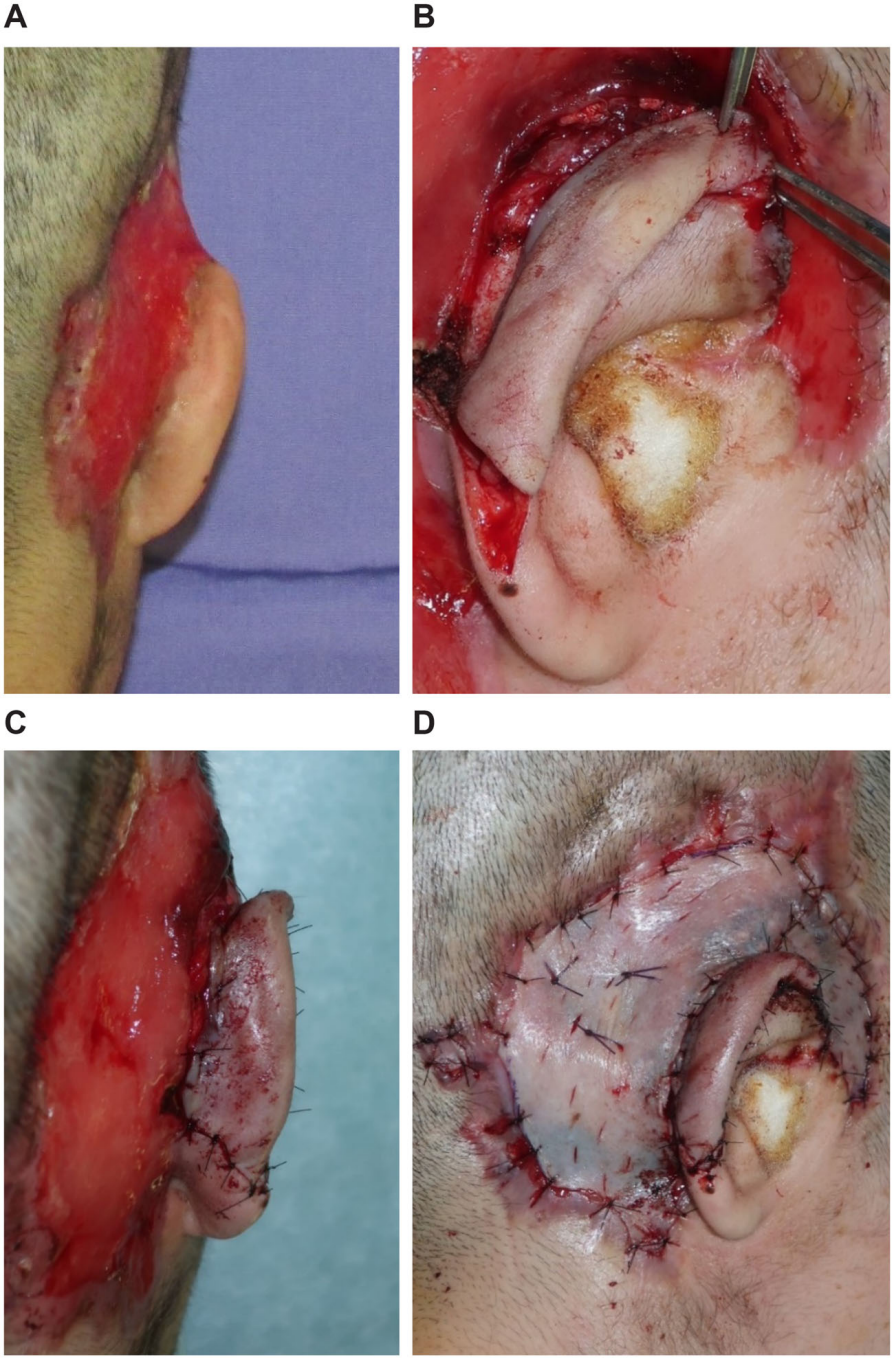
Reconstructive surgery, 66 days after. (A) Preoperatively, the auriculotemporal sulcus has disappeared. (B) VY advancement flap in the auricle. (C) The auriculotemporal sulcus is created. (D) Skin grafting on the temporal area.

**Figure 6.**
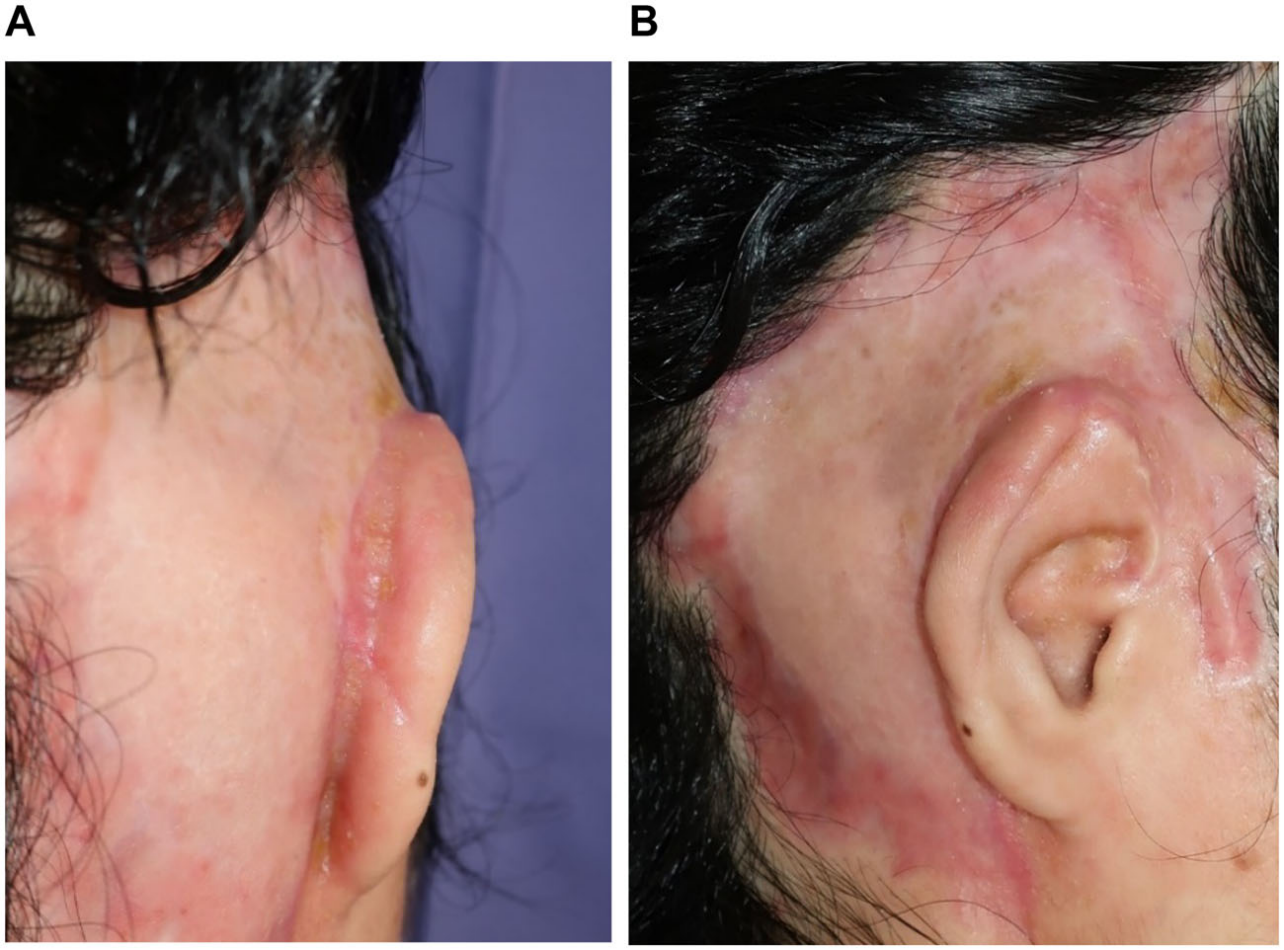
At 7 months after reconstructive surgery. (A) The auricular temporal sulcus is clear. (B) The shape of the auricle is good.

At 66 days after the first debridement, the patient underwent reconstructive surgery by creating an auriculotemporal sulcus and skin grafting. The upper auriculotemporal sulcus region was incised, the VY advancement flap was created in the auricular region, and the exposed auricular cartilage was covered by the flap. The ulcer surface of the temporal region was covered with a split-thickness skin graft from the parietal region (Figure 5). The patient was discharged from the hospital 41 days after the reconstructive surgery. After 7 months, the auricle and graft were provided with better outcomes (Figure 6). Moreover, the patient can wear a mask.

## Discussion

NSTI has been classified in various ways based on the pathology and site of lesions, and the superficial fascia is often the main site of infection and necrosis, known as ‘necrotizing fasciitis’ [4]. However, since the treatment strategy is similar and requires control of the source of infection, such as debridement, the use of the term ‘NSTI’ has been proposed as a comprehensive term for the disease [5].

Only a few reports of NSTI in the auricle and temporal region have been reported. In the head and face, reconstruction is difficult due to the limited skin and soft tissues. In the present case, the infection extended to the superficial and deep temporal fascia, which were necrotic. The fascia has lesser blood flow than the muscle and is more likely the primary site of infection; however, because the fascia is a tough tissue that simultaneously protects the muscle, the underlying muscle may be uninfected. Hence, although the infection was severe that the upper part of the auricle was detached, the temporalis muscle was protected by the superficial and deep temporal fascia, and the infection did not extend to the temporalis muscle.

Generally, the auricular region reconstruction often involves the use of local skin flaps made from the adjacent skin, temporoparietal fascia, or mastoid facia [6–9]. Therefore, the temporalis flap was selected to salvage the auricle, which was partially separated from the temporal region as much as possible. The muscular flap is resistant to infection as the tissue with abundant blood flow and is thought to be advantageous in covering exposed tissues with poor blood flow [10]. Conversely, the disadvantage of this approach is that if a temporary reconstruction is performed using adjacent tissues in the extreme phase of infection, the tissue may indeed be infected or necrotic. In the present case, the temporalis muscle below the superficial and deep temporal fascia, the primary site of infection, was not necrotic due to infection based on the appearance, such as color tone, and findings, such as petechial hemorrhage. As infection and inflammation in the surrounding tissues have not been completely controlled by debridement, inflammation may spread to the periosteum because of the exposed temporalis muscle, resulting in partial cranial bone exposure.

The scalp reconstruction methods for exposed cranial bone include local skin flaps, tissue expander techniques, and, in extensive cases, free flap using the latissimus dorsi muscle, or the greater omentum may also be an option [11]. Two-stage skin grafting has also been indicated using an artificial dermis with an ingenious method of excising the outer plate, even in extensive scalp defects [12]. In the present case, the exposed extracranial plate was shaved until the occurrence of petechial hemorrhage, covering the artificial dermis, and NPWT was used in combination. The combined use of an artificial dermis and NPWT has been reported to promote the formation of granulation and increase the rate of artificial dermis implantation [13,14]. The wound was closed by skin grafting. The disadvantages of using the temporalis flap and achieving wound closure by skin grafting are bald hair and depressed deformity; however, in the present case, the patient could be concealed with hair.

## Conclusions

We have described a case of NSTI around the right auricle in which half of the right auricle was detached from the temporal region by debridement; however, the auricle was salvaged by creating the temporalis muscle flap. The auricular reconstruction was significant not only in terms of appearance but also in function, such as the ability to wear a mask. In cases of severe infection, the temporalis muscle flap may be an option for salvaging the auricle as much as possible.

## Patient consent

Consent for publication was obtained from the patient.
